# Overcoming mental health stigma through student’s awareness and project-based inclusive pedagogy in English teaching colleges: Moderating role of institutional support in China

**DOI:** 10.3389/fpsyt.2022.992904

**Published:** 2022-10-20

**Authors:** Yuan Gao

**Affiliations:** ^1^Zhengzhou University of Technology, Zhengzhou, China

**Keywords:** mental health stigma, mental health awareness, project-based learning, inclusive pedagogy, institutional support, effective English teaching, constructivist theory

## Abstract

Mental health stigma undermines collaborative work and creates communication breakdowns for students to face real-world challenges. Moreover, project-based English learning in East-Asian nations is a significant challenge for advancing students’ effective learning, while students lack mental health self-awareness. Unawareness causes distractions and results in learning inefficiencies. Furthermore, without institutional support (IS) achieving effective learning could never be possible. Therefore, this study investigates the relationships between project-based learning (PBL) and mental health awareness (MHA) with effective English language teaching among university students in China. We also tested the moderation effect of IS in the relationships between PBL and MHA with effective English language learning (EEL). Using the purposive sampling technique, we collected valid responses from 713 students studying English as a foreign language in universities and colleges in Harbin, China. The findings interestingly supported the direct hypotheses between PBL and EEL, as well as MHA and EEL. Moreover, the moderating role of IS established a significant effect on both PBL and EEL, as well as MHA and EEL, respectively. Policymakers, practitioners, and academicians should understand that integrating PBL as an inclusive pedagogy method with proper IS may enhance effective learning; however, it will consume more time.

## Introduction

English teachers have been facing severe criticism in East Asian economies, as they must transform their teaching methodologies into an inclusive pedagogy to promote students’ effective learning (1, 2). Educators have been experimenting with various methods for effective English teaching in East Asian nations to guarantee that every student in their class has an equal opportunity while enjoying learning English. Academicians have developed many terms like “learning for knowledge” to explain the learning and its methodologies that depend on the student’s understanding (1). Among these methods, few refer to minor changes in methodology, whereas most require teachers to seek, evaluate, and explore the students to develop their ideas. In such dynamic learning perceptions, awareness is of utmost importance, and eventually, it grows when students and teachers are actively involved in discussions. Previous research has highlighted that students’ effective learning depends on good acoustics (2). For instance: digital literacy (3), students’ competence (4), and the role of a teacher (5). In ineffective teaching methods, students face severe issues in comprehending the class content leading to underperformance (6). It creates problems in learning not only language-related four skills of listening, speaking, reading, and writing, but it also deteriorates other cognitive, affective, and psychomotor skills to be used in the subject matter. Moreover, it establishes mental health-related challenges for the students (7). The present study aims to check the relationship between the students’ mental health awareness (MHA) and practical learning. One significant challenge is measuring the extent of students’ MHA in light of specific mental health concerns and effective learning.

Knowing the students’ best learning approaches and how they affect their learning direction makes these approaches crucial. The constructive basis for learning is influenced by how well individuals know about their mental health, as MHA activities reinforce students’ perceptions of learning. For effective learning to occur, collaborative efforts from academic institutions and their personnel in providing students an environment of learning, inspiration, and equal opportunities to learn must be considered most of the time, students who are interested in learning ultimately mainly participate in classroom activities is the outcome good mental health. So, the good mental health of an individual student can turn him/her into a significant contributor to the learning environment, facilitate the effective learning process of their classmates, and be an enormous help to the campus community. However, regrettably, none of these possible advantages are always reaped.

Due to widespread COVID-19 disease worldwide, every country has made significant decisions to ensure their public health safety (8). COVID-19 has also brought a tsunami of information, from which most are misleading rather than mischief (9). This misleading information led to disobedience at a community level, and the public did not follow the health safety-related protocols set by the government. This makes it even more significant for the public to have health-related awareness (8). It is connected with mental health and needs special attention because common health-related understanding causes severe cognitive health issues (10). According to van den Broucke (11), there must be strict guidelines for sharing any news related to COVID-19 because the cycle of fake news has caused depression, anxiety, and other psychological issues (12). If the situation persists, this will significantly affect the mental health quality of the individuals (13).

Fitri (14) noted that putting much study-related burden on the students upsets their mental health, and they go through a cognitive and emotional burden. Mental health in China needs earnest attention because about 12 out of 1,000 people reported having mental health-related issues (15). It is against the national plans to promote mental health among individuals (9). The most vulnerable group is those ranging from 15 to 24 years, and most of these are students at a school/college or a university (14). A survey by Beasley et al. (16) mentioned that 39% of college students face mental health-related issues. A study by Chai et al. (15) concluded that 43.7% of the students who participated in the study had experienced anxiety. At the same time, 22.6% had experienced depression due to a COVID-19 pandemic and class-related matters. Hence, it is crucial to conduct research to explain and measure MHA among students and its impact on their practical learning. Moreover, it is also important to note what institutional support (IS) students have to become aware of their mental health through mental health literacy programs, and to what extent it influences the relationships between project-based learning (PBL), MHA, and effective English language learning.

The development of a country depends upon the quality of its academic institutions, and the COVID-19 pandemic has opened up new avenues for academic institutions to teach online and use PBL methods of teaching, which incorporate the use of technology, develops teamwork in the students by providing them enough time for discussion the assigned topics, and thoroughly understanding the topics. As a result, academic institutions must also develop systems to enhance academic performance (17). For students’ effective learning, improvement in knowledge, creativity, opting for new teaching methods, and student participation are the key elements (18). According to Piaget’s constructivist theory (19), learning occurs when individuals are stimulated toward interactions with others and get involved in social communication to pursue their knowledge development. Hence, to promote group learning, bringing fascinating and suitable content for class activities, the role of teachers becomes more significant because it enhances the learning capacity of students, and they learn more effectively (20). PBL enhances effective learning by introducing the subject videos and related projects in the class by which students can learn more (21). For effective learning of students, the teachers must be equipped with the necessary professional knowledge. Education is now replacing the orthodox teaching methods and using more novel approaches to give students hands-on experience with the subject (22). Therefore, student-centric teaching methods increase students’ interest in learning and achieving the learning objectives, increasing students’ learning satisfaction (23).

Therefore, the current study aims to investigate the direct and indirect relationships between the variables empirically and proposes the below-given research questions (RQs): RQ1. What is the direct impact of PBL and MHA on EEL? RQ2. To what extent IS is related to the EEL of the students, and is the relationship between PBL, MHA, and EEL moderated by IS? The study primarily contributes to the literature by clarifying PBL from an educational standpoint. Secondly, our study investigates the effects of MHA on students’ effective learning. The study provides empirical evidence that PBL and MHA positively affect students’ effective learning. Finally, this paper examines the moderating role of IS between PBL, MHA, and effective English language teaching.

## Literature review and hypotheses development

### Constructivist theory

Piaget (19) was the first scholar to introduce constructivism in academia, and the concept was based on cognitive development. This theory is based on the premise that people learn through observation and scientific techniques and then develop their understanding of the world around them. According to Piaget’s constructivist theory (19), learning occurs when individuals are stimulated toward interactions with others and get involved in social communication to pursue their knowledge development. There are three basic requirements of constructivist theory, (1) The teachers’ teaching methodology reflects effective learning. (2) Effective learning occurs when individuals adopt reality and behave as expected. (3) Effective learning occurs due to one’s autonomous control over his/her mental health. Whereas Dtiscoll (24) described that knowledge could only exist in human knowledge, and it does not require to be matched with the realities of the world, humans keep updating this knowledge through their life experiences. Constructivist theory stimulates students’ natural curiosity about the world around them and how things work. Constructivist learning theory is known to have three major streams. These three streams are individual, social, and collectivist learning streams. Our study uses the constructivist theory as an underpinning theory and develops the framework using individual learning.

### Inclusive pedagogy in English language teaching

Student-centric teaching approaches focus on student learning, which results in effective learning. On the one hand, it is a very dynamic process and can take the forms of formal and informal learning. On the other hand, dull teaching methods, unchanging content, and lack of professional skills are also very static. It is a fact that learning develops awareness, morality, behavior, capabilities, and preferences about life. According to Forsey et al. (25), the process through which student gets the opportunity to participate in high-quality learning is known as practical learning. In high-quality education systems, results are connected to well-defined quantifiable learning standards that encourage students’ learning. Students’ effective learning is also measured when the instructors implement their vital academic objective targets and evaluate students within a specific timeframe (26). Such an evaluation system not only facilitates assessing their achievements and improving their effectiveness but also leads toward expanding educational programs (27). Such outcome-based education facilitates teachers and students in developing a shared understanding of the course’s and program’s learning objectives.

Specific technical aspects in the e-learning network, such as cognitive, instructional, and social representation, have been utilized to measure effective learning. Such design components foster high-level cognitive skills *via* deep learning and research. Effective learning addresses questions: “Am I able to recall the material I have learned? Am I able to explain the material I have learned?” Therefore, a learner’s capacity to openly express what they have learned *via* quantitative methods is called effective learning. Prior studies have mentioned that learning brings changes to the advancement of students learning (25). In this study, learning outcomes are related to direct effects on learning through PBL (1).

### Project-based learning and effective English language teaching

Besides the fact that scholars do not agree on a single definition of PBL, its advocates still agree on some of its fundamental aspects (28). According to Loyens et al. (29), the PBL method is a common collaborative and research-oriented learning strategy distinguished by active interaction with students and their relative learning. Based on the purpose it serves, PBL is divided into four categories: making a final product (Producer’s Project); knowing a subject and enjoying its knowledge or experience (Consumer’s Project); improving a specific technique or skill (Specific learning); solving a philosophical problem (Problem Project) (30). Kokotsaki et al. (31) mentioned that students taught using the PBL approach must work closely to find an optimum solution to a problem, perform new product development for a defined audience, and then assess the project and its development process. The usage of the PBL method is a requirement of the modern age because it cultivates critical thinking skills, develops problem-solving, enhances interpersonal communication skills, leadership skills, teamwork through collaboration among the members, media literacy, and most importantly, encourages students to be more creative while proposing the solutions (32). A study by Mettas and Constantinou (26) examined the effects of PBL on students and teachers and found that PBL improves problem-solving competence. Another research found that PBL increased academic performance in both (33).

Research by Lavy and Shriki (34) concluded that PBL positively affects learning perception. Furthermore, an evaluation process is helpful for student instructors since it assists them in developing a clear understanding of their learning objectives, which can improve the educational development of the participants they supervise. Which is followed by the creation of opportunities for the students in the form of support in project selection, to study the complex issues, interact with a problem with complicated solutions, search for viable solutions, evaluate the patterns, and critically review their work for revisions, and finally coming up with a generic solution. In his research on teaching languages, Miller (35) mentioned that teachers using PBL as a teaching method are encouraged to obtain multiple instructional objectives like enhancing communication skills, mixing cultural studies with linguistics, developing relationships between the teaching material and the language, and comparing the pupils’ first and the second languages. These assigned projects could be as small as just a selection of a research topic or as big as an exhibition, wall painting, theater performances, and dramas (36). Students’ effective language learning through PBL is supported by many studies (1, 25).

A study by Rohmahwati (37) investigated the PBL effects on the students’ speaking ability, and the results mentioned that implementing the PBL method has positively increased the students’ speaking ability. Moreover, the students of the speaking class also depicted a positive attitude toward the PBL implementation. Additionally, Marwan (38) did action research with 25 students from a vocational higher education institution, using PBL with information and communication technologies in an English classroom. The study’s findings revealed that pupils become more motivated to utilize English. Furthermore, students had more fascinating and relevant learning in an English PBL session. Based on all the arguments mentioned above, we formulate the below-given premise.

H1: Project-based learning positively influences EEL teaching.

### Mental health awareness and effective English teaching

The ability of an individual to cope with stressful situations, work efficiently, and contribute to society for betterment. World Health Organization (39) has termed this state of wellbeing as mental health. MHA plays a vital role in building the capacity to achieve and maintain a constructive psychological state in dealing with difficulties and challenges of daily life. Improving MHA entails teaching people how to recognize the signs and symptoms of mental illness, which is essential for seeking help, receiving treatment, and implementing preventative measures (40). Keyes (41) model of MHA includes three components: psychological, social, and emotional wellbeing, and concluded that mental health is an individual’s capacity to recognize and assess his/her affective states and psychological and social functioning. Howell (42) conducted an exploratory study to evaluate the student’s learning in the university and consider psychological wellbeing as the MHA factor. It is because psychological wellbeing describes the approach to living and functioning effectively and actualizing human potential (i.e., Eudaimonia) (43), which is especially crucial to university functioning. It encompasses many Eudaimonia-related notions, such as self-acceptance, positive relationships with others, personal progress, life purpose, autonomy, environmental mastery (44), and relatedness, competence, engagement, and meaning (45).

Individuals facing mental health-related challenges are on the verge of disconnection from their daily activities, becoming less productive, more often having health concerns, and having a higher probability of being involved in an unforeseen event (46, 47). Preventive initiatives in avoiding mental health disorders are essential public health approaches for reducing mental disorders’ health, social, and economic impact (48). Academic institutions possess the best intervention settings and can play the best part in it due to the fact the students spend most than half of the day studying at their academic institutions, and in case they seek any help or need any advice about their mental health issues, the administration and faculty/staff will be their first point of contact (49). Faculty or staff of academic institutions can easily approach students suffering from higher-order mental issues (50) and assess overall mental health services (51). Academic institutions can play a vital role in providing opportunities for MHA in the students because the absence of required awareness and knowledge about mental health issues can cause early mental health stigma in students, and they usually link this mental disorder with conflict and violence (52). Consequently, academic institutions must make efforts to increase the MHA of their students, which can boost their awareness and understanding of mental diseases and help in their detection, management, or prevention to improve their academic performance (53). Based on all the arguments mentioned above, we formulate the below-given premise.

H2: Mental health awareness positively influences EEL teaching.

### Institutional support as a moderator

According to LaMastro (54), the support service that academic institutions provide to the students to nurture and value contributions is called IS. From the compelling learning perspective, IS refers to the active support and assistance provided by institutions in the form of rules, regulations, and financial and non-financial assistance that motivate students to carry out their obligations in a highly effective and efficient manner. Research conducted by Rhoades (55) explained that individuals get motivated on a volunteer basis to embrace the IS system, and Celep and Yilmazturk (56) confirmed that IS does affect commitment. A study investigated the impact of IS on teacher responsiveness and student satisfaction and performance, discovering that IS had a favorable influence on student performance and satisfaction (57).

**FIGURE 1:**
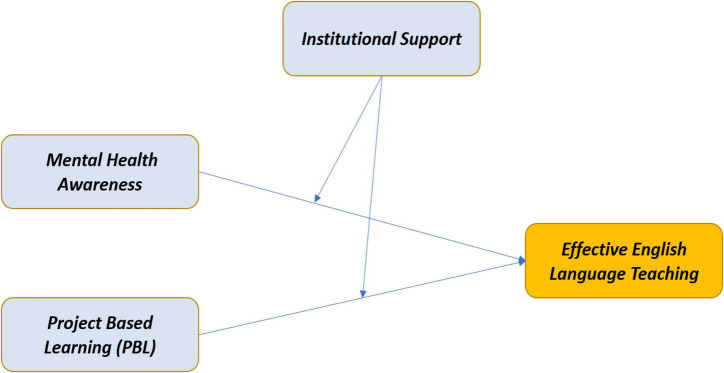
Theoretical framework.

People become confident after believing they receive continuous IS and achieve their performance goals (58). Along the same line, Yildirim et al. (59) emphasized a need for training and IS for using learning management systems in educational institutions. One important thing to be noticed is that access to a help desk, which is functional, user-friendly, and provides all the necessary support and guidance, can make learning exciting and compelling (60, 61). If handled effectively, it can assist in establishing enduring communal bonds between students, improving students’ learning results. Students and teachers require a supportive atmosphere regarding PBL, excellent facilities, resource access, and equipment utilization. Based on the findings of Abdullah and Primus (62) regarding the dearth of specific indigenous support centers in Malaysian public universities, institutional assistance substantially impacts student participation. It is, therefore, proposed that students taught using PBL as a teaching method and awareness of mental health are more likely to perform better if there is institutional and organizational support. Figure 1 shows the research model of the study.

H3: Institutional support moderates the relationship between PBL and EEL, so the positive relationship will be stronger when the IS is high.

H4: Institutional support moderates the relationship between MHA and EEL, such that the positive relationship will be stronger when the IS is high.

## Methodology

It is explanatory research that employs a quantitative data-gathering strategy. The information was gathered from schools, colleges, and universities. The data was collected using a survey approach and a survey questionnaire. Prior to data collection, respondents were educated. There were two sections to the questionnaire. The respondent’s demographic information was obtained in the first part. In the second portion, we offered questions about each of our variables. The response to each question item was obtained using a five-point Likert scale. The non-probability purposive sampling approach was used to acquire data from the students who are learning English not only as a mandatory subject but also learning it abroad for higher education. The information was gathered from 713 respondents. SPSS was used for fundamental analysis, while AMOS v.24 was used for structural equation modeling (SEM) to test the hypothesis.

### Questionnaire design

The questionnaire in this study was designed on a five-point Likert scale. This form of questionnaire is said to help gather data from a large population in a trustworthy manner. Indeed, a survey-based questionnaire is vital for collecting data from respondents since it is simple to disseminate and collect the questionnaire for analysis. As a result, the current study used the same data gathering strategy. The scale questions were collected from past studies with careful attention and face validity to gather data from respondents. The research adopted a nine-item measure for MHA from the study of Rush and Grouzet (63).

Further, the six scale items for students’ effective English language teaching were adopted from the study of Tang and Chaw (64). In the same way, the 10 scale items for PBL were adopted from the study Shin (65). Lastly, the three scale items for IS were adopted from the study Abdullah and Primus (62). In this way, the scale item for MHA was taken to determine the relationship between MHA and effective English language teaching for the students. Understanding that MHA plays a critical role in people’s mental development and awareness is critical. Also, these scale items were previously used by different studies to understand the role of mental health education in the better performance of the student’s in-class activities.

Similarly, the scale questions for students’ MHA were carefully chosen to recognize the importance of students’ psychological wellbeing. To adequately quantify the link, this study used scale items that were carefully chosen. Furthermore, the IS scale items were used to assess the moderating function of IS in the link between PBL, MHA, and successful English learning in Chinese students. Finally, the scale items for PBL were used to collect data to understand its relationship to Chinese students’ effective English language learning. Furthermore, the expert’s opinions were considered when testing the face validity of these scale items, and the research experts were contacted to provide their opinions on the face validity of the questionnaire. As a result of receiving positive responses from various experts, these scale items were incorporated into the questionnaire to effectively collect data from the target respondents.

### Data collection process

In this section of the study, the detail of the data collection process is presented. Firstly, the current study respondents were students of different schools/colleges/universities in Henan province of China. Therefore, the non-probability purposive sampling technique was adopted, and students were provided with the questionnaire. Notably, the respondents’ consent was taken to respond to the questionnaire. Further, with the positive response from the individual students who were learning English to go abroad, the questionnaire was distributed to them, and they were provided with a brief introduction to the study to get familiarity with it. Also, the individuals were allowed to ask any question related to the study, with difficulty responding to the questionnaire. In this regard, 750 questionnaires were provided to the students with the technique of random sampling, as it is appropriate to collect the data from a large population. Similarly, 713 questionnaires were taken back from the students and analyzed for this study with a response rate of 95%. Finally, the students were thanked for their precious time and contributed to the study by the researcher.

## Findings

### Respondents’ demographic

This study researched students learning English at different schools/colleges/universities in Henan province of China, so all the respondents were students. Table 1 shows the demographic characteristics of the respondents. Data were collected from 10 schools, 20 senior high schools, and 10 universities in Henan province, China, where 61.3 percent of the students were 18–23 years of age, 12.9 percent were from the 24–29 age group, and 14.4% were from 30 to 35 years of age. 70.1 percent of the respondents were male students. 72.7% of these respondents were undergraduate students, and 27.3% mentioned that they were postgraduate students. Of our total of 713 respondents, 32.7% mentioned studying English for 1.5–2 years, whereas 29.9% studied for only 6 months. Most respondents (62.4%) had less than two weekly lectures, whereas only 9% took daily classes. Lastly, 71.5% of the respondents had an English class inside the campus, and 16.5% had classes outside the campus. Lastly, to our question about current mental health, surprisingly, 27.2% mentioned poor mental health at the time of data collection.

**TABLE 1:** Respondents’ demographic characteristics.

Variable	Categories	Frequency	Percentage
**Gender**			
	Male	500	70.1
	Female	213	29.9
**Age**			
	18–23 years old	437	61.3
	24–29 years old	92	12.9
	30–35 years old	103	14.4
	36–40 years old	34	4.7
	>40 year old	23	3.3
		24	3.4
**Level of education**			
	Undergraduate	416	72.7
	Postgraduate	195	27.3
**Experience of learning English**			
	0–6 months	213	29.9
	6 months to 1 year	127	17.8
	1–1.5 years	87	12.2
	1.5–2 years	233	32.7
	More than 2 years	53	7.4
**Frequency of English lectures per week**			
	Less than 2 times a week	445	62.4
	3–4 times a week	200	28.1
	Daily	68	9.5
**Level of English study**			
	Foundation	246	34.5
	Diploma	213	29.8
	Bachelor’s degree	174	24.4
	Master’s degree	57	8
	Doctoral degree	23	3.3
**Location status of English institute**			
	Hometown or outside campus	118	16.5
	Inside campus	509	71.5
	Others	86	12
**Current health status**			
	Poor	194	27.2
	Good	519	72.8

### Reliability, validity, and measurement model tests

The convergent validity of the constructs in the present study was addressed based on the criteria suggested by Hair et al. (66). These criteria are: (1) the factor loadings for the measurement items of all the constructs must exceed 0.60, (2) the value of composite reliability for each of the constructs must be equal to or larger than 0.70, and (3) the value of the average variance extracted (AVE) for each of the constructs is more extensive than 0.50. Further, an item from the resistance construct had been removed due to its low loading (i.e., less than 0.60). After removing such items, the outputs in Table 2 have shown that the measurement model fulfilled the requirements of convergent validity. Therefore, convergent validity has been conclusively established for this study.

**TABLE 2:** Convergent validity, reliability and factor loadings.

	Scale Items	Loading	AVE	CR	Cronbach’s α
Project-based learning (PBL)			0.586	0.927	0.932
	PBL1	0.723			
	PBL2	0.813			
	PBL4	0.788			
	PBL5	0.786			
	PBL6	0.783			
	PBL7	0.799			
	PBL8	0.759			
	PBL9	0.845			
	PBL10	0.835			
Mental health awareness (MHA)			0.531	0.879	0.886
	MHA1	0.918			
	MHA2	0.682			
	MHA3	0.742			
	MHA4	0.621			
	MHA5	0.627			
	MHA7	0.865			
	MHA9	0.847			
Institutional support (IS)			0.854	0.662	0.851
	IS1	0.855			
	IS2	0.870			
	IS3	0.883			
Effective English language learning (EEL)			0.588	0.847	0.856
	EL1	0.761			
	EL2	0.711			
	EL3	0.705			
	EL4	0.803			
	EL5	0.800			
	EL6	0.804			

There was no multicollinearity issue in the data set as the variance inflation factor (VIF) values were between 1.004 and 1.142, and tolerance values ranged between 0.876 and 0.999, as suggested by Hair et al. (67), which is an apparent absence of multicollinearity. In addition, the discriminant validity for the present study was assessed using the guideline proposed by Fornell and Larcker (68), which is based on comparing the squared root of AVE values against the maximum shared variance (MSV) values of its own and the variance of other constructs. The discriminant validity results highlighted in Table 3 show that the values of the squared root of AVE (diagonal entries in bracket) are smaller than the values of MSV on their own and more remarkable than the variance shared between any two constructs (off-diagonal entries in italic).

**TABLE 3:** Discriminant validity.

	MSV	MHA	PBL	IS	EEL
MHA	0.08	0.729			
PBL	0.08	0.283***	0.766		
IS	0.014	0.051	0.118†	0.814	
EEL	0.02	0.012	−0.143*	0.052	0.699

Significance of correlations: †p < 0.100; *p < 0.050; ***p < 0.001.

### Structural equation modeling

The next step is to check the causal model. We first run the SEM on our basic model. At first, the model was not fit, and the results were abysmal. Then, to ensure that the data has no poor fit values, follow the recommendations by Hair et al. (67) and re-estimate the model fitness values. Table 4 shows the model fitness test’s initial model and specified values. Furthermore, the results are evident after the modified model is fit for further analysis.

**TABLE 4:** Measurement model fitness values.

CFA indicator	Threshold value	Initial model	Modified model
CMIN/DF	≤3	5.29	1.908
GFI	≥0.80	0.835	0.87
AGFI	≥0.80	0.776	0.841
CFI	≥0.90	0.913	0.95
RMSEA	≤0.08	0.1	0.056
NFI	≥0.90	0.913	0.901
TLI	≥0.90	0.896	0.943
IFI	≥0.90	0.914	0.95
PCLOSE	>0.05	0.000	0.080
SRMR	<0.08	0.091	0.064

### Hypothesis testing

**FIGURE 2:**
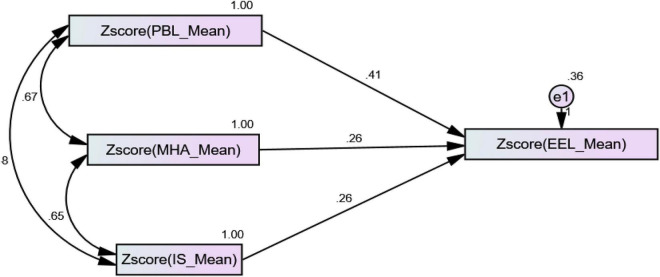
Structural equation modeling (SEM) results of direct effects.

We first checked the direct effects of our proposed hypothesis, as shown in Figure 2.

The first hypothesis is a relationship between PBL and students’ effective English language learning. Moreover, the estimated values show a positive relationship between PBL and EEL with the values (β = 0.406 and *p* < 0.001). The second hypothesis states a relationship between MHA and EEL, and values (β = 0.262 and *p* < 0.001) show a positive relationship. All the direct hypothesis results are shown in Table 5.

**TABLE 5:** Direct path effect coefficients.

Hypothesis	Structural relationships	Coefficient (β)	Standard error	t Statistics	*p*-value
H1	ZPBL → ZEEL	0.406***	0.042	9.963	0.000
H2	ZMHA → ZEEL	0.262***	0.049	5.366	0.000
H3	ZPBL → ZEEL	0.559***	0.036	15.432	0.000
	ZIS → ZEEL	0.471***	0.04	11.736	0.000
	ZPBL_x_ZIS → ZEEL	0.203***	0.03	5.304	0.000
H4	ZMHA → ZEEL	0.551***	0.045	12.183	0.000
	ZIS → ZEEL	0.402***	0.049	8.148	0.000
	ZMHA_x_ZIS → ZEEL	0.221***	0.036	5.106	0.000

PBL, project-based learning; MHA, mental health awareness; IS, institutional support; EEL, effective English language learning.

**FIGURE 3:**
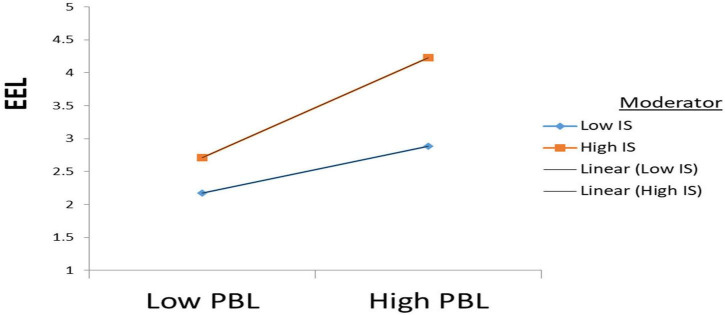
Moderation results (H3).

**FIGURE 4:**
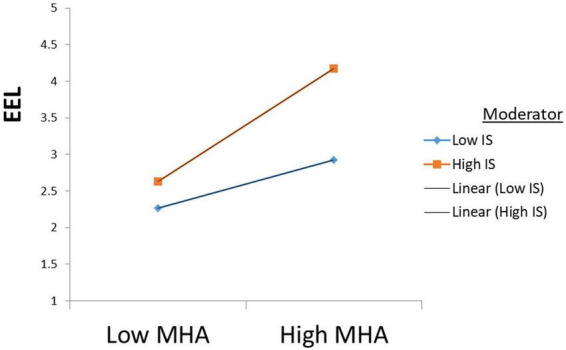
Moderation results (H4).

Furthermore, according to the results, IS moderates the positive relationship between PBL and EEL (β = 0.203, *t* = 5.304); therefore, H3 is accepted, see Figure 3. Also, according to the results, IS moderates the positive relationship between MHA and EEL (β = 0.221, *t* = 5.106). Hence H4 is also accepted see Figure 4.

## Discussion

The current study aims to develop a new way of thinking to improve students’ ability to learn English effectively. This project aimed to use the PBL approach with high school, college, and university students. The relationships between the PBL and EEL were examined in light of the proposed framework. Keeping the study objectives in mind, we examined the usage of the PBL teaching methodology among the students learning English and tried to understand its effects on their effective learning. The results of our empirical research indicate that the usage of PBL in teaching increased effective English language learning in the students. The results indicate that the PBL method builds English language-related capabilities in students. Students have acquired much English-related information in the form of vocabulary, learned how to use grammar structures, developed relationships with their classmates due to the class and off-class conversations, and finally developed an understanding that participation in PBL makes learning easier. As also noted by Häkkinen et al. (32) that PBL approaches make students ready to face the challenges in their daily life and perform better under demanding situations. Our findings also align with (69–71), who mentioned that developing students’ understanding using PBL methodology improves the educational learning of media and information and thus improves their academic performance.

Additionally, the findings of this study are empirical evidence showing that the teachers and students of this era consider PBL an essential element. PBL ensures that students can manage their academic workload and improves their learning environment simultaneously. The connection in the PBL approach helps students achieve their academic objectives, simplifies things, and preserves the relationship between students and professors.

In order to better understand students’ approaches to achieving academic goals, another objective of the study was to test the relationship between MHA and EEL. According to this study, students having MHA said they had succeeded in their goals. According to the findings above, students with solid MHA can adjust to their surroundings, and MHA sparks a drive to improve themselves favorably. Additionally, those with good MHA have intense emotional, personality, and physical traits that enable them to adjust to any stressors that may arise in their surroundings. The findings indicate that individuals with mental awareness realize they are competent and can perform classroom activities, feel energized while interacting in class, and quickly adapt to new situations. They are aware of whatever they are doing. These findings align with Granlund’s results (72), as they explained that individuals with good MHA not only harness the potential to develop the abilities to cope with the pressures of life and perform better, but their contribution to the community gets improved.

The MAH is all about initiating preventive actions in case of any mental problem identification and, to manage it, taking instant control over it for better handling without being ashamed. One easy way to be literate about mental health-related matters is to acquire knowledge and information through media about several disorders, management, and restraints for mental health-related issues. Fitri (14) mentioned that individuals with the MHA and capability to maintain adequate self-mental health status. Therefore, MHA goes beyond simply grasping the basics of MHA; instead, it is also about convictions necessary to cultivate favorable attitudes about the significance of mental wellness (73).

This study also examined IS influencing relationships between PBL and MHA. The study found that IS helps individuals with relatively good PBL and MHA. If the level of IS increases, the relationship between PBL and EEL strengthens. Simultaneously, the strength of the relationship between MHA and EEL increases. The findings unmistakably imply that IS mechanisms can significantly promote students’ effective English language learning. The results indicated that academic institutions provide support services like communities to make friends, learn new skills, enjoy campus life, perform volunteer activities, peer support, mentorship programs, academic support services, and lecture support in terms of advice from academic staff/faculty.

Consequently, enhancing effective student learning requires more IS that could be monetary (scholarships, funding, stipends etc.) or non-monetary (academic counseling, clubs, societies, student community centers, volunteer services etc.) must be provided available and easily accessible to all students. Our research findings match those of previous researchers (55, 57, 58). The positive influence of IS was found to develop an online learning system of education (59).

## Implications

### Theoretical implications

This research utilized beneficial tools to identify the effects of PBL and MHA on EEL. Researchers can keep looking for measurement-related errors when reviewing the literature on effective learning. Learning will be complex for unfamiliar, puzzled, unwelcoming, or unsupportive students in the classroom. Different pertinent factors that affect how well students learn may be covered in other schools. The study contributes to the literature by clarifying PBL from an educational standpoint. Secondly, our study investigates the effects of MHA on students’ effective learning. The study provides empirical evidence that PBL and MHA positively affect students’ effective learning. Results also indicated that PBL plays a vital role when it comes to the student’s effective learning. Finally, this paper examines the moderating role of IS between PBL, MHA, and effective English language teaching.

### Practical implications

Project-based learning requires students to solve issues together with their peers. PBL allows students to share knowledge (i.e., peer learning). Maraj (74) reported that many students feel that learning from others increases their grasp of the subject being studied. It is evident from this scoping study that PBL gives students the skills and knowledge necessary for successful PBL and efficient learning. According to studies, positive academic accomplishment is correlated with solid cooperative abilities, MHA, and institutional assistance (75, 76). The moderation result directs policymakers in China to pay heed to provide adequate IS to learners and instructors. The effects of PBL and MHA in the classroom on efficient student learning were investigated in this study. The study’s findings might benefit learners by fostering comfort, healthy development, and simple learning. In order to support all children’s academic growth, we also looked at research on how educators may respond to various degrees of adaptation, handle adversity, and develop resilience. Likely, learning styles do not exist in how their proponents see them, making it hard to recognize them in pupils and instruct them accordingly. By working in groups and using PBL, students learn more. Some respondents said they were dealing with mental health issues and that this was affecting their academic performance. According to the previous study, academics can better plan their class activities to prevent and minimize the many mental health difficulties that students may experience by realizing that such issues impair students’ learning (74). Another option for policymakers is creating a channel to support students who offer volunteer activities. Student clubs and societies can offer instruction, encourage learning, and generate opportunities for their peers.

## Limitations and future directions

This scoping review aims to document current PBL practices that are effective. It was highlighted that given the new finding on effective learning behavior, a sizable number of articles within this period were determined to be pertinent to PBL. The study’s sample is one potential factor that could restrict the results’ external validity. In our sample, five universities in Harbin, Heilongjiang, were chosen, but there is no clear target demographic. The fact that we only evaluated the one form of PBL extensively covered earlier in the text represents another study limitation. There may be significant differences between this instantiation and other people’s ideas of PBL.

Additionally, we focused on generally healthy senior citizens in the community, which may have limitations on generalizability and practical uses. The study employed a cross-sectional methodology and only collected data at one point, often seen as less accurate than data acquired from the same respondents several times. Thus, conducting longitudinal data analysis in subsequent research would be preferable. Future studies on the most efficient learning strategies to apply in academic situations should consider the opinions of instructors and other higher education partners. Advice on how to integrate the learning method into other areas of learners’ learning stages in the future should be given to teachers. Future studies may give further insight into addressing the problem in academic settings. Researchers can keep looking for measurement-related errors when going through the literature on effective learning, and they can also add other factors into the framework, like game-based learning and cooperative learning (1). Future studies can also study it from cognitive and social perspectives (41). Furthermore, we did not use the demographic information for any deep analysis; future studies can use such respondents’ characteristics to conduct group analysis and take deep insights.

## Conclusion

This study showed a positive relationship between PBL, students’ MHA, and effective learning. Although PBL has a direct positive effect on students’ effective learning and IS, it has a more significant effect on their performance in formal classes. Furthermore, the role of IS in the relationship between students’ MHA and their effective learning is also significantly and positively strengthened when IS is increased. PBL approaches in academic settings, based on our findings, develop and improve the abilities of students to learn effectively. It also develops cooperation with group members, teamwork spirit, and ideas sharing in group settings. PBL also is equally essential for the teachers as it facilitates the teachers in establishing a better understanding of the student’s academic situation. Academic institutions may be able to take advantage of the resources already there in the student body itself and stop more long-term suffering by changing the way they view this as a “detriment” or “vulnerability” to campus mental health efforts and instead of viewing it as a “strength” in these efforts. Additionally, this study is the first to our knowledge that offers a model linking MHA and EEL through the moderating role of IS for effective learning. In this regard, academic institutions should deliberately maximize IS as it increases their degree and learning.

## Data availability statement

The raw data supporting the conclusions of this article will be made available by the authors without undue reservation. Requests to access the datasets should be directed to the corresponding author (YG) at 20141010@zzut.edu.cn.

## Ethics statement

The present study was conducted in accordance with the Declaration of Helsinki and was reviewed/approved by the Ethical Committee for the Zhengzhou University of Technology, Zhengzhou, China. All study participants provided a signed version of informed consent to participate.

## Author contributions

The author confirms being the sole contributor of this work and has approved it for publication.
